# A Soluble NK-CAR Mediates the Specific Cytotoxicity of NK Cells toward the Target CD20^+^ Lymphoma Cells

**DOI:** 10.14336/AD.2022.0415

**Published:** 2022-10-01

**Authors:** Rongjiao Liu, Qizhi Luo, Weiguang Luo, Ling Wan, Quan Zhu, Xiangli Yin, Xiaofang Lu, Zixuan Song, Leiyan Wei, Zhiqing Xiang, Yizhou Zou

**Affiliations:** ^1^Department of Immunology, School of Basic Medical of Central South University, Changsha, Hunan, China.; ^2^Department of Laboratory Medicine, Henan Provincial People's Hospital; People’s Hospital of Zhengzhou University, Zhengzhou, China.; ^3^Hunan Key Laboratory of Aging Biology, Xiangya Hospital, Central South University, Changsha, China.

**Keywords:** Soluble NK-CAR, NKG2D, MICA, ScFv, NK cells targeted cytotoxicity

## Abstract

The structures of chimeric antigen receptors (CARs) currently designed for natural killer (NK) cells are mostly based on knowledge gained about CAR-T cells. Although these CAR-NK cells have shown promising effects, there are still many limitations to their application. In this study, we designed a soluble NK-CAR since the membrane protein NKG2D expressed by NK cells can directly trigger NK cell cytotoxicity by binding with the ligand MICA. This CAR is composed of three segments: the extracellular domain of MICA, an anti-CD20 single-chain variable fragment (anti-CD20 ScFv), and a human IgG Fc component. The nucleotide sequence of the soluble NK-CAR was cloned into a eukaryotic expression vector and expressed in suspension HEK293 cells, and the recombinant NK-CAR protein was then purified in a *Staphylococcus aureus* protein A column. The novel NK-CAR exhibited bifunctional activity, recognizing both the CD20 antigen of target cells and the NKG2D receptor of NKL cells. The NK-CAR activated the NKG2D receptor signaling pathway, causing NKL cells to express CD107a and secrete interferon-gamma. The soluble NK-CAR mediated the NKL cell killing of CD20^+^ Daudi cells in vitro, with a 1 µg/mL concentration inducing the maximum killing effect. Moreover, 51.7% (p < 0.01) of Daudi cells were killed at the effector-to-target ratio of 10:1. In the presence of recombinant rMICA and NKG2D-Ig proteins, this killing effect was reduced to 30% (P < 0.01) owing to competitive interference. Our results highlight the clinical application potential of this novel immunotherapy for killing target tumor cells.

In recent years, the excellent outcomes of chimeric antigen receptor (CAR) T-cell therapy for hematological tumors have opened a new path for cancer therapy [1, 2]. However, this therapeutic option still has certain disadvantages. For example, aside from being a complex and time-consuming process for individualized treatment, it can also cause cytokine release syndrome [3] and graft-versus host-disease (GVHD) [4]. Another limitation of CAR T-cell therapy is its poor treatment of solid tumors, which is mainly attributed to physical barriers and the tumor microenvironment (TME) [5, 6].

Natural killer (NK) cells are derived from pluripotent hematopoietic progenitors in bone marrow [7]. As an important member of the innate lymphocytes, NK cells have a similar cytotoxic mechanism to that of T cells in killing target cells, which gives them a vital role in the immune surveillance for tumor cells [8]. The concept of CAR-NK cell therapy is gaining attention because NK cells are less likely than T cells to induce GVHD and can kill target cells through antibody-dependent cellular cytotoxicity [9]. To date, the design of most CAR structures for NK cells have been based on those of CAR-T cells [10, 11]. Although these CAR-NK cells have antitumor activity [12-14], they still face similar limitations to those of CAR-T cells in that they can only be applied on an individualized therapy basis and are restricted by the TME of solid tumors. Therefore, the development of new CARs for NK cell-based immunotherapy is of particular importance [15-17].

The natural killer group 2, member D (NKG2D)-NKG2D ligand (NKG2DL) axis is the major pathway through which human NK cells are activated to kill tumor cells and virus-infected cells [18-20]. As an important receptor on NK cells, NKG2D triggers the activation and cytotoxicity of these cells by binding to its cognate ligand on target cells [21]. NKG2D is expressed on the NK cell surface as a homodimer, and its cytoplasmic region does not contain the immunoreceptor tyrosine-based activation motif (ITAM) but couples to the YxxM motif-containing DAP10 homodimer to transmit the activation signal [22]. Then, p85 phosphoinositide 3-kinase and the Vav-1-Grb2 complex are recruited to initiate the activation of a series of downstream pathways for activating the NK cells. Subsequently, the activated NK cells release perforin and granzymes or produce cytokines, such as interferon-gamma (IFN-γ), to kill the tumor cells [23]. NKG2D has multiple ligands, including the MHC class I chain-related protein (MIC) and UL16-binding protein (ULBP1-6) families [24]. The MIC isoforms MICA and MICB are the most well-studied NKG2DLs [25-28] and have been reported to effectively activate NK cells after binding to NKG2D [29, 30].

In this study, we designed a soluble NK-CAR on the basis of the NKG2D-NKG2DL axis theory. This soluble receptor, designated as MS-Ig, contains the MICA extracellular domain (MIC-ECD), an anti-CD20 single-chain variable fragment (anti-CD20 ScFv), and a human IgG crystallizable fragment (IgG Fc) arranged in order from the N-terminal to the C-terminal ends (i.e., MICA-anti-CD20 ScFv-Fc). In the single-chain antibody of MS-Ig, the anti-CD20 ScFv component binds to the CD20 antigen on cells of B-cell lymphoma, whereas the MICA-ECD binds to the NKG2D receptor on NK cells to directly activate their killing of the cancerous B-cells.

## MATERIALS AND METHODS

### Cell lines and culture

The MICA^+^ Hmy2.CIR, Daudi, H9, and NKL cell lines were from stocks held in our laboratory. The NKG2D-2B4 and 2B4 cells were licensed for use by the Chengcheng Zhang Laboratory of the University of Texas Southwestern Medical Center (TX, USA). All of the cell lines listed above were cultured in RPMI 1640 basic (1×) medium (Cat#: 11875500BT, Gibco) containing 10% fetal bovine serum (FBS; Cat#: 10099141, Gibco). The medium for the NKL cells also contained 10 ng/mL interleukin-2 (Cat#: 200-02-50UG, PeproTech). All cells were cultured at 37°C in incubators containing 5% CO_2_. Additionally, suspension HEK293 cells were cultured in dedicated and serum-free SMM 293-TII medium (Cat#: M293TII, Sino Biological Inc.).

### Construction of the recombinant expression vectors

The nucleotide sequence of MS-Ig was synthesized by the Beijing Genomics Institution. and the MS-Ig fragment was cleaved with the restriction endonucleases HindIII (Cat#: R3104V, NEB) and BamHI (Cat#: R3136V, NEB) and ligated into the eukaryotic expression vector pFlag-CMV5.1 using T4 ligase (Cat#: EL0011, Invitrogen). After transfecting the ligase product into competent *Escherichia coli* DH5α cells, the recombinant cell colony was amplified, and the plasmids were extracted for sequencing verification. The plasmid with the correct sequence was chosen as the pFlag-CMV5.1-MS-Ig recombinant vector. The other recombinant plasmid used in this study, pFlag-CMV5.1-MS, had already been constructed earlier in our laboratory and contains the MS-Ig sequence without the IgG Fc component.

### Expression and purification of the fusion protein

The constructed recombinant plasmid was mixed with the transfection reagent polyethylenimine (25 kDa linear PEI, Cat#: 23966, Polysciences Inc.) at the plasmid (µg)-to-PEI (µL) ratio of 1:3, for 15 min at 25°C. Then, suspension HEK293 cells were transfected with the mixture at a final concentration of 1 µg/mL. The culture supernatant was collected on days 3 or 4 after transfection and diluted to a ratio of 1:4. Then, the protein in the supernatant was aggregated on a *Staphylococcus aureus* protein A (SPA) column (KONCEN), eluted with PBS of pH 2.6, and neutralized using PBS of pH 9.8. The protein was concentrated on an ultrafiltration column (Ultra-4, Amicon) and stored at -80°C. The MS protein was expressed using a similar method to that used for MS-Ig and was then labeled with 6×His and purified via Ni-ion affinity chromatography (Bestchrom) using 0.5 M imidazole-containing phosphate buffer as the eluent (Cat#: 288-32-4, Sigma).

### Coomassie brilliant blue staining

For separation of the protein by 5% sodium dodecyl sulfate (SDS)-free polyacrylamide gel electrophoresis (PAGE) and 10% SDS-PAGE, 4× loading buffer was added to the denatured protein and the sample was placed in boiling water for 5 min. The natural protein was mixed with 6× loading buffer without SDS. Then, electrophoresis of the denatured and natural protein was performed. After electrophoresis, the gels were stained with Coomassie brilliant blue dye (Cat#: R-250, Sigma) for 30 min at 25°C and decolorized overnight.

### Western blot analysis

Following the electrophoretic separation of the proteins as described above, the protein bands were transferred to a polyvinylidene difluoride membrane, after which nonspecific sites on the membrane were blocked with 5% skim milk at 37°C for 1 h. After blocking, the primary antibody was added, and the membrane was incubated at 37°C for 1 h and then washed three times with 0.05% Tris-buffered saline (TBS)-Tween. Next, the membrane was incubated with either horseradish peroxidase (HRP)-conjugated anti-mouse IgG Fc (Cat#: 115-035-071, Jackson Immuno Research Labs) or HRP-conjugated anti-human IgG Fc (Cat#: 109-035-098, Jackson Immuno Research Labs) secondary antibodies at 37°C for 30 min and then washed 3 times with 0.05% TBS-Tween. Finally, an enhanced chemiluminescence reagent was added to visualize the results.

### Flow cytometry

The cell lines to be tested were respectively centrifuged, after which 2 × 10^6^ cells were resuspended in 5% FBS-containing PBS and incubated with the specific antibody at a 1:100 ratio. For CD20 detection, the primary antibody used was phycoerythrin (PE)-conjugated anti-human CD20 antibody (Cat#: 12-0209-41, eBioscience). For MICA detection, the anti-human MICA antibody (Production in our own laboratory) was added first, followed by PE-conjugated anti-mouse IgG (H+L) antibody (Cat#: 1034-09, SouthernBiotech), and the control was the cells with only anti-mouse IgG Fc antibody added. For CD3 detection, PE-conjugated anti-CD3 antibody (Cat#: 344816, Biolegend) was added to H9 cells only. For NKG2D detection, PE-conjugated anti-NKG2D antibody (Cat#: 320806, Biolegend) was added to NKL cells. For CD107a detection, PE-conjugated anti-CD107a antibody (Cat#: 328608, BioLegend) was added to NKL cells. The isotype antibody was PE-conjugated anti-mouse-IgG1 (Cat#: 400113, Biolegend). All antibodies were incubated with the cells for 30 min at 4 °C and then washed twice with 5% FBS-containing PBS. Finally, a flow cytometer was used to evaluate the cells.

### Flow cytometric analysis of MS-Ig binding

In brief, MS-Ig at a concentration of 10 µg/mL was added to 2 × 10^6^ cells to be tested and incubation was carried out for 30 min at 4°C. After incubation, the cells were centrifuged and then washed twice with and resuspended in 5% FBS-containing PBS. Next, PE-conjugated anti-human IgG Fc antibody (Cat#: 12-4998-82, eBioscience) was added to the cells at 1:100 ratio for incubation at 4°C for 30 min. For the control group, only PE-conjugated anti-human IgG Fc antibody was added to the cells (i.e., no MS-Ig was added). Finally, after two further washes with 5% FBS-containing PBS, the cells were evaluated using a flow cytometer.

### NKG2D receptor activation and detection

NKG2D-2B4 cells or 2B4 cells were adjusted to 5 × 10^4^ cells/mL, and 200 µL was then added to each well of a 96-well plate. The recombinant protein was added to a final concentration of 10 μg/mL and the plate was incubated for 16 h at 37°C under 5% CO_2_. Green fluorescent protein (GFP) luminescence was detected using a fluorescence microscope. For flow cytometric analysis of the activation of NKG2D-2B4 cells, the concentrations of MS-Ig and anti-CD20 antibody tested were 0, 0.5, 1, 5, and 10 μg/mL, and that of the competitive binding-blocking proteins (rMICA and NKG2D-Ig) was respectively 10 μg/mL.

### NKL cell activation and detection

The wells of 96-well plates were coated with 10 μg/mL MS-Ig and the plates were incubated at 37°C for 4 h. Thereafter, the wells were gently washed twice with PBS, following which 2 × 10^5^ NKL cells in 100 μL of medium were added to each well. For the control, soluble MS-Ig was added directly to NKL cells. For activation of the NKL cells via co-incubation of the soluble MS-Ig with target cells, 100 μL each of 2 × 10^4^ H9 cells, MICA^+^ HMy2.CIR cells, and Daudi cells was added into the respective wells of 96-well plates. All plates were then incubated at 37°C for 4 h under 5% CO_2_. Finally, the culture supernatant was collected, diluted 1:1, and subjected to enzyme-linked immunosorbent assay for IFN-γ using a commercial kit (Cat#: CHE0017, 4A BIOTECH Co., Ltd.).

### Cytotoxicity test

After staining with carboxyfluorescein succinimidyl ester (CFSE; CellTrace CFSE Cell Proliferation Kit, Cat No. C34554, Fisher Scientific), the target cells were added into the wells of 96-well plates (5 × 10^4^ cells/well). Then, NKL cells were added at different effector-to-target (E:T) ratios (i.e., 1:1, 5:1, 10:1, and 25:1). After co-culturing of the cells at 37°C for 4 h, the apoptotic cells were stained with 7-aminoactinomycin D (7-AAD; Cat#: 51-68981E, BD Pharmingen) and evaluated using flow cytometry. The killing efficiency was calculated by analyzing the proportion of 7-AAD-positive CFSE-stained target cells (CFSE^+^ 7-AAD^+^), with the CFSE-stained target cells set as 100%. The protein concentration gradient used was 0, 0.01, 0.1, 1, 5, and 10 μg/mL.

### Statistical analysis

All experimental results are based on at least three independent replicate experiments. The data are shown as the means ± standard deviation, and statistical analyses were performed using GraphPad Prism 7. The percentage data were analyzed with the chi-squared test. The quantitative data were determined to be normally distributed according to the Kolmogorov-Smirnov test and were analyzed using the independent-samples t-test. A P value of less than 0.05 was considered to indicate statistical significance.

## RESULTS

### MS-Ig is a soluble homodimer fusion recombinant protein

**Figure 1.**
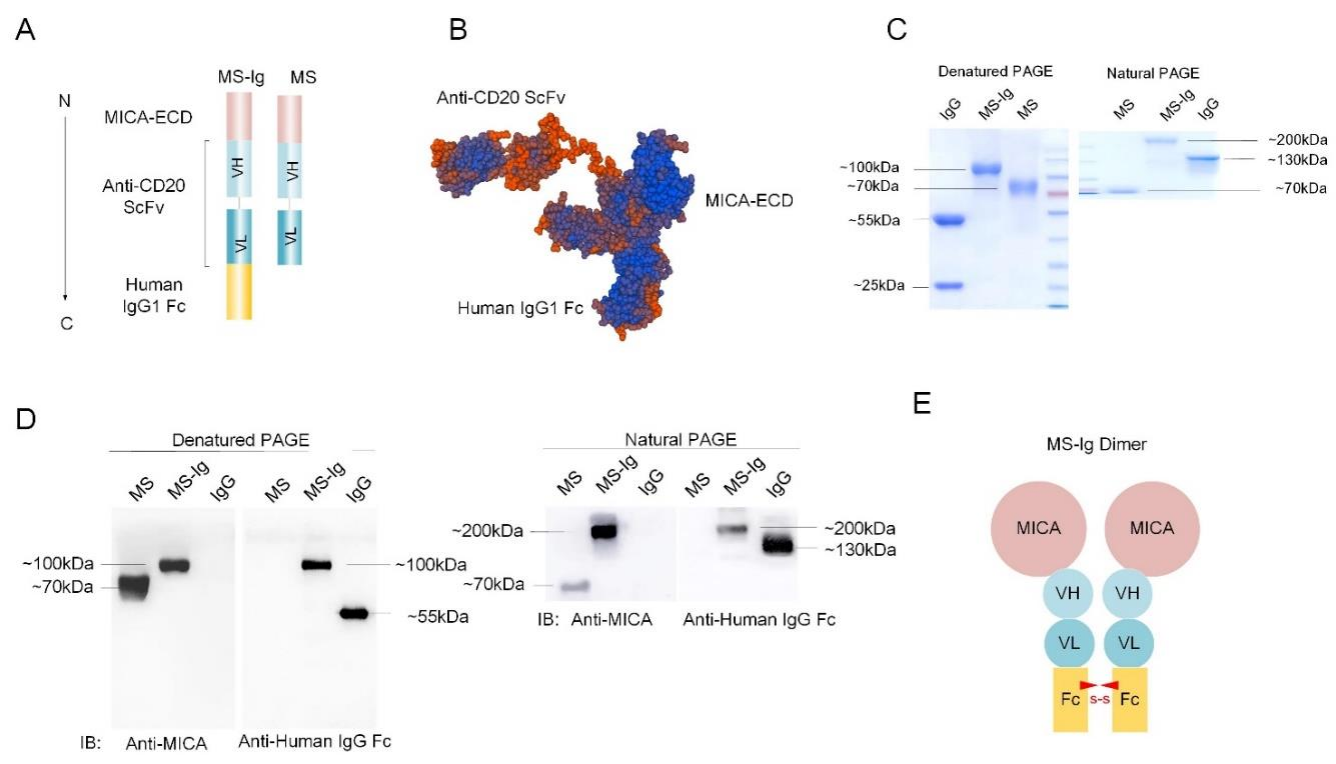
**Design and present form of the soluble NK-CAR (MS-Ig)**. (**A**) MS-Ig design. MS is the control recombinant protein without IgG Fc. (**B**) Three-dimensional structural model of the MS-Ig protein monomer, created on the SWISS MODEL website through input of the amino acid sequence. (C, D) Coomassie brilliant blue staining and western blot analysis of MS-Ig. MS and IgG were used as controls. The gel concentrations for the denatured PAGE and natural PAGE were 10% and 5%, respectively. The images of the natural PAGE were compressed for more a convenient comparison. (**E**) Diagrammatic sketch of the MS-Ig dimer.

MS-Ig consists of three parts: the MICA-ECD, an anti-CD20 ScFv, and a human IgG Fc component arranged in order from the N-terminal to the C-terminal ends, respectively. The recombinant MS protein lacking the human IgG Fc component was used as the control. The anti-CD20 ScFv contains the variable regions of a heavy chain (VH) and a light chain (VL), which are connected by a (GGGGS)_3_ linker. The MICA-ECD was designed to bind with the NKG2D receptor of NK cells, whereas the anti-CD20 ScFv was designed to bind with the CD20 antigen on the target cells (Fig. 1A). The three-dimensional structure of MS-Ig was predicted using the algorithm available on the SWISS MODEL website, where the best prediction result demonstrated the clear partitioning of the three components, with no hindrance of one another’s space (Fig. 1B). Next, the full MS-Ig sequence was artificially synthesized and inserted into the eukaryotic expression vector pFlag-CMV5.1 to construct the pFlag-CMV5.1-MS-Ig recombinant vector for subsequent transfection into suspension HEK293 cells. The recombinant MS-Ig protein was easily purified on an SPA column. Coomassie brilliant blue staining (Fig. 1C) and western blot (Fig. 1D) analyses of MS-Ig in both the denatured and natural states were performed, with MS used as the control. According to the results, the molecular weight of natural MS-Ig is approximately 200 kDa, almost twice that of its denatured counterpart (100 kDa). By contrast, both the natural and denatured MS proteins are of the same size (70 kDa), indicating that the MS-Ig protein is a homodimer in its natural state (Fig. 1E).

### MS-Ig has dual bioactivity of binding with the CD20 antigen of target cells and the NKG2D receptor of effector cells

**Figure 2.**
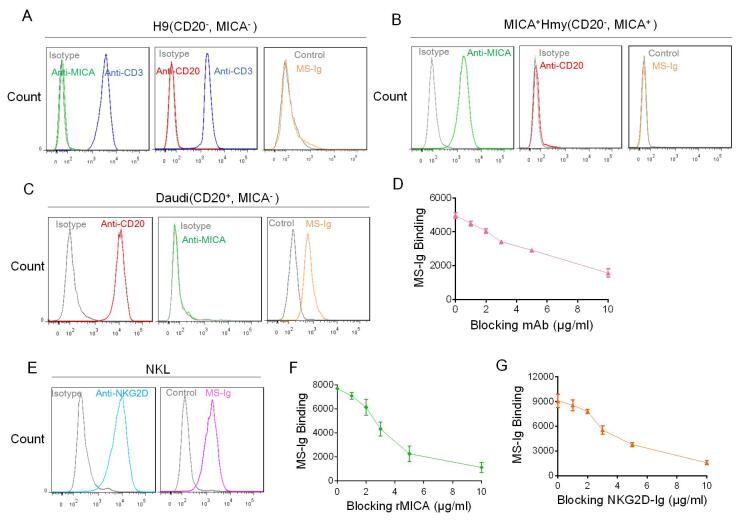
**MS-Ig binds specifically to target and effector cells**. (A-C) Flow cytometry results of the expression of CD20 and MICA on H9 cells (CD3^+^, CD20^-^, MICA^-^), MICA^+^ HMy2.CIR cells (CD20^-^, MICA^+^), and Daudi cells (CD20^+^, MICA^-^) and their binding to MS-Ig. (**D**) Flow cytometry results of the binding changes of MS-Ig to Daudi cells following the addition of the concentration gradient of blocking mAb (anti-CD20). The mean fluorescence intensity (MFI) value represents the assessment index of MS-Ig binding. (**E**) Flow cytometry results of the expression of NKG2D of the NKL cells and its binding to MS-Ig. (F, G) Flow cytometry results of the binding changes of MS-Ig to NKL cells following the addition of the concentration gradient of competitive binding-blocking proteins rMICA and NKG2D-Ig.

To evaluate the bioactivity of MS-Ig, its ability to bind to both target cells and effector cells at the same time was validated. In this study, Daudi cells expressing CD20 were selected as the representative of B-cell lymphoma, and NKL cells expressing the NKG2D receptor were representative of NK cells. First, flow cytometry was used to confirm that the H9 cells were negative for CD20 and MICA and positive for CD3 (CD20^-^MICA^-^CD3^+^) (Fig. 2A); that the HMy2.CIR cells stably expressing MICA (MICA^+^ HMy2.CIR) were negative for CD20 (CD20^-^MICA^+^) (Fig. 2B); and that the Daudi cells were negative for MICA but positive for CD20 (CD20^+^MICA^-^) (Fig. 2C). Additionally, the binding of MS-Ig with the Daudi cells (CD20^+^MICA^-^) only was also confirmed (Fig. 2C). To verify that MS-Ig was bound specifically to CD20 on the Daudi cells, a concentration gradient of anti-CD20 antibodies was added for blocking the antigen-binding site. As a result, the binding between MS-Ig and the Daudi cells decreased gradually with increasing anti-CD20 antibody concentration, indicating that the binding of the recombinant NK-CAR protein to the target cells was specifically via the anti-CD20 ScFv component (Fig. 2D). Next, using flow cytometry, we verified the expression of NKG2D on the NKL effector cells and showed that MS-Ig could bind effectively to these cells (Fig. 2E). We also tested the specific binding of MICA by adding recombinant MICA (rMICA) protein, which binds competitively with the NKG2D receptor on NKL cells. The binding between MS-Ig and the NKL cells weakened gradually with increasing rMICA concentration (Fig. 2F). When soluble NKG2D fusion protein (NKG2D-Ig) was added, the binding of NKL cells with MS-Ig weakened gradually with increase in the competitor protein concentration (Fig. 2G). These results indicate that the binding of MS-Ig to NKL cells involved the specific binding between the MICA-ECD and NKG2D.

### Immobilized form of MS-Ig can activate the NKG2D receptor signaling pathway in reporter cells

**Figure 3.**
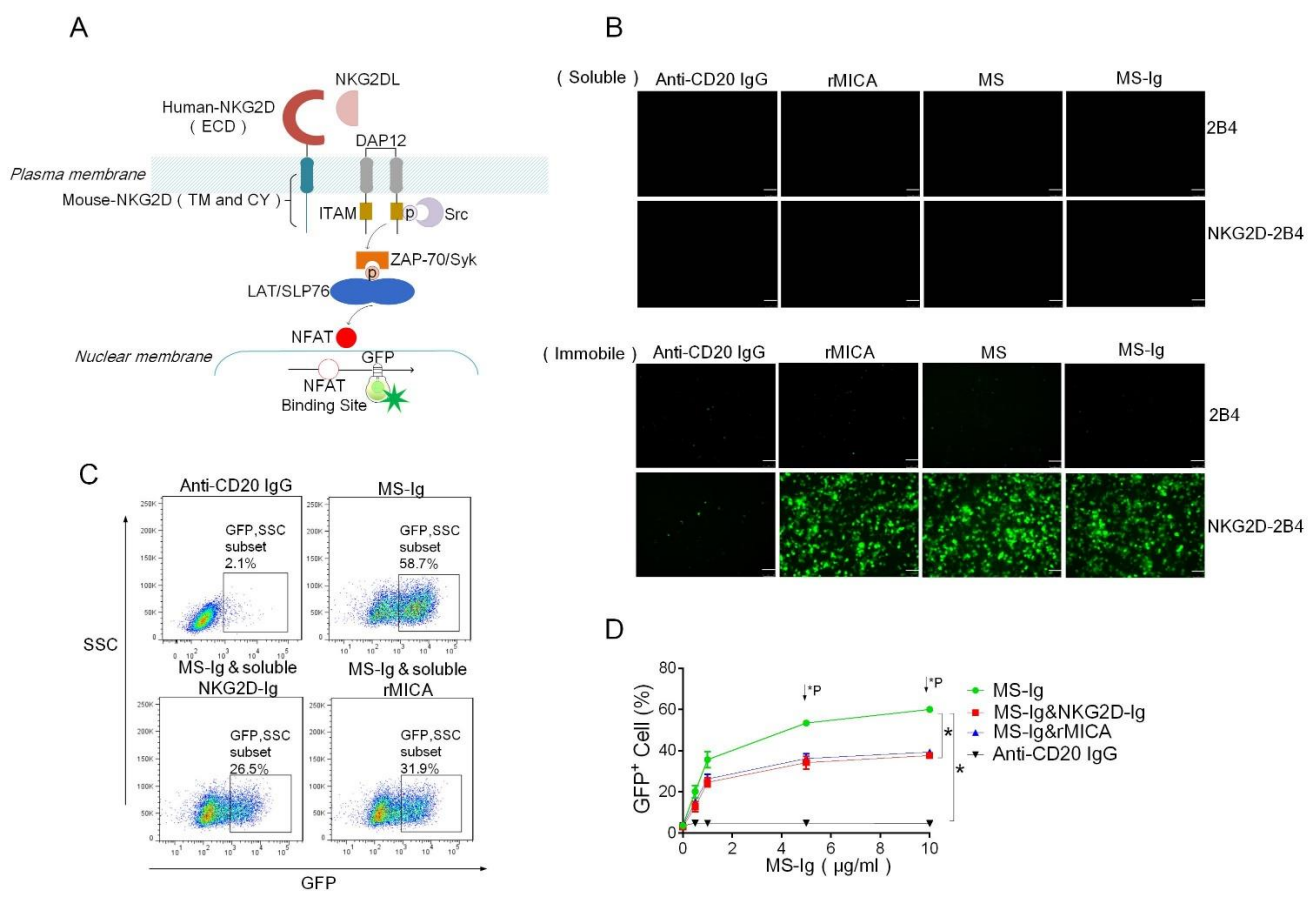
**MS-Ig has the ability to activate the NKG2D receptor signaling pathway**. (**A**) Composition of the human and mouse chimeric NKG2D-2B4 reporter system. (**B**) Fluorescence microscopy images of NKG2D-2B4 cells and 2B4 cells after their incubation with immobilized MS-Ig for 16 h. Anti-CD20 IgG, rMICA, and MS were used as the controls. (**C**) Flow cytometry results of the percentage of GFP^+^ NKG2D-2B4 cells after the co-incubation of NKG2D-2B4 cells with immobilized MS-Ig in the presence of soluble binding-blocking proteins rMICA and NKG2D-Ig. Anti-CD20 IgG was used as the control. (**D**) Calculated percentages of GFP^+^ NKG2D-2B4 cells in the presence of MS-Ig at various gradient concentrations and in the presence of the binding-blocking proteins rMICA and NKG2D-Ig. *P < 0.05, based on the chi-squared test.

After confirming that MS-Ig can bind to both the CD20 antigen of target cells and the NKG2D receptor of effector cells, its ability to activate the NKG2D receptor signaling pathway needed to be verified. On the basis of the methods of a previous study [31], MS-Ig was coated onto and thereby immobilized in cell culture plates, and NKG2D receptor reporter cells (NKG2D-2B4) were then added. This human-mouse chimeric NKG2D receptor reporting system integrates the human NKG2D extracellular domain, the mouse NKG2D-S transmembrane region, and an intracellular region into mouse 2B4 cells. The chimeric NKG2D receptor can bind to human NKG2DLs, while the transmembrane and intracellular regions of mouse NKG2D couple to DAP12. The ITAM in DAP12 is phosphorylated by the Src family of serine kinases, whereupon zeta-chain-associated protein kinase-70 (ZAP-70)/tyrosine-protein kinase (Syk) are recruited and phosphorylated, leading to the induction of the linker for activation of T cell (LAT) and lymphocyte cytosolic protein 2 (SLP-76). These latter proteins then activate the biochemical events downstream of the recruiting signaling molecules to induce the activation of calmodulin and nuclear factor of activated T cells (NFAT), ultimately inducing the expression of the GFP gene in 2B4 cells, whereupon the NKG2D-2B4 cells emit green fluorescence (Fig. 3A).

**Figure 4.**
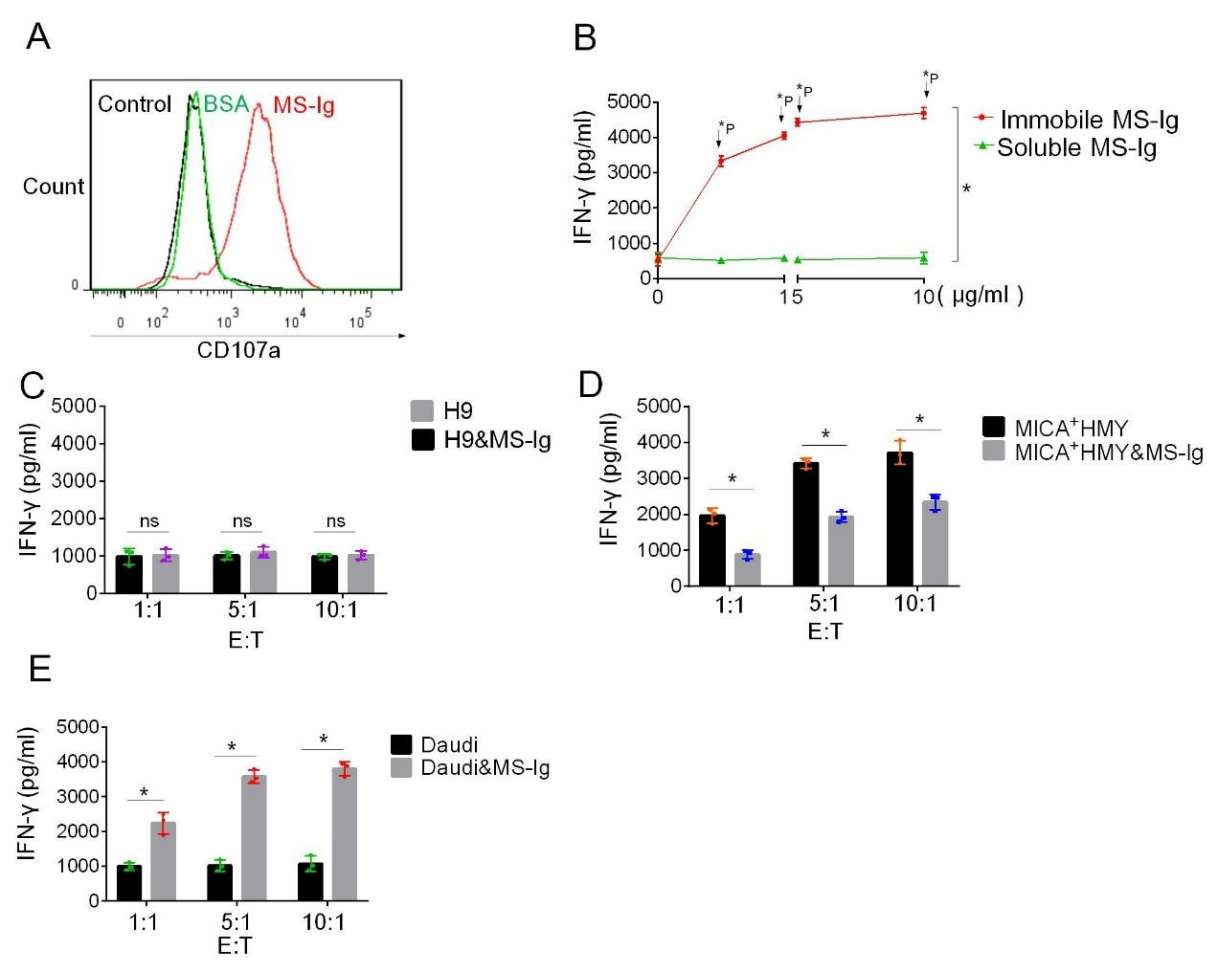
**MS-Ig activates NKL cells**. (**A**) Flow cytometry results of the expression of CD107a on NKL cells co-incubated with immobilized MS-Ig. The isotype antibody was used as the control. (**B**) ELISA measurements of the amounts of IFN-γ produced by NKL cells co-incubated with immobilized and soluble MS-Ig, respectively. (C-E) Concentrations of IFN-γ detected in the culture supernatant of H9 cells, MICA^+^ Hmy2.CIR cells, and Daudi cells co-incubated with MS-Ig at different effector-to-target (E:T) ratios together with NKL cells at the same time. *P < 0.05, based on the independent-samples t-test.

NKG2D-2B4 cells were treated with either the soluble or immobilized forms of the anti-CD20 IgG, rMICA, MS, and MS-Ig proteins. Neither the NKG2D-2B4 cells nor the 2B4 cells were activated by the soluble proteins, indicating that soluble MS-Ig can bind with the NKG2D receptor but cannot active the NKG2D receptor pathway. Among the four immobilized proteins, anti-CD20 IgG was the only one that could not activate NKG2D-2B4 cells, and all four of them had no effect on 2B4 cells without NKG2D expression (Fig. 3B). These results suggest that the MICA-ECD of immobilized MS-Ig has the function of activating the NKG2D receptor signaling pathway. We further investigated the activation of NKG2D-2B4 cells with MS-Ig using flow cytometry. After the reaction system had been blocked with 10 μg/mL soluble rMICA or NKG2D-Ig, the percentage of GFP-positive reporter cells decreased from 58.7% to 31.9% and 26.5%, respectively (Fig. 3C). Additionally, there was a dose-effect relationship between the concentration of MS-Ig and the activation of NKG2D-2B4 cells. The percentage of activated cells was reduced significantly from 60.1% to 39.3% (χ^2^ = 8.8, P < 0.01) after the addition of 10 μg/mL soluble rMICA, and from 60.1% to 7.7% (χ^2^ = 9.7, P < 0.01) after the addition of 10 μg/mL NKG2D-Ig (Fig. 3D). These data indicate that immobilized MS-Ig has the functional role of activating the NKG2D receptor signaling pathway.

### NKL cells can be activated by the soluble MS-Ig fusion protein when bound onto CD20^+^ target cells

Having determined that immobilized MS-Ig can specifically activate the NKG2D receptor signaling pathway, we used it to perform activation experiments on NKL cells that express high levels of NKG2D, where the levels of CD107a expression and IFN-γ release were used for evaluating cell activation. Upon the co-incubation of immobilized MS-Ig with NKL cells, the flow cytometry results showed that the expression of CD107a was increased (Fig. 4A). The concentrations of IFN-γ released by the NKL cells were also increased with increasing concentration of immobilized MS-Ig, with the peak IFN-γ concentration reaching 4600 pg/mL (Fig. 4B). By contrast, IFN-γ was hardly detected in the system with soluble MS-Ig added. These results were consistent with those of the NKG2D receptor reporter cells, in that immobilized MS-Ig could activate the cells whereas soluble MS-Ig could not. These findings further confirmed that soluble MS-Ig can bind to the NKG2D receptor but has no ability to activate the NKG2D receptor signaling pathway in NKL cells, a capability that only immobilized MS-Ig has.

Next, the ability of soluble MS-Ig to activate NKL cells after binding with target cells was examined. Soluble MS-Ig was added respectively to H9, MICA^+^ HMy2.CIR, and Daudi cells, and the IFN-γ concentration in each cell culture supernatant was determined. Regardless of the absence or presence of MS-Ig, there was no difference in the concentration of IFN-γ (<1000 pg/mL) in the H9 cell reaction systems (Fig. 4C). By contrast, when MICA^+^ HMy2.CIR cells were used as the target cells, the concentration of IFN-γ reached 4000 pg/mL in the absence of MS-Ig (E:T ratio of 10:1). However, in the presence of MS-Ig, the IFN-γ concentration was significantly reduced from 4027 ± 137.3 pg/mL to 2712 ± 83.41 pg/mL (t = 6.0, P < 0.01) (Fig. 4D). Because of the binding between the MICA-ECD of soluble MS-Ig and the NKL cells, the binding of MICA^+^ HMy2.CIR cells with the NKL cells was partially blocked, and NKL cell activation was subsequently weakened. When soluble MS-Ig was added to Daudi cells, the NKL cells were stimulated to release IFN-γ, the levels of which increased from 1046 ± 141.8 pg/mL to 4204 ± 117.8 pg/mL (t = 15.5, P < 0.01) at the E:T ratio of 10:1 (Fig. 4E). This indicated that soluble MS-Ig could bind to Daudi cells to form an immobilized state—similar to the membrane-type MICA on HMy2.CIR cells—thereby activating the NKL cells.

**Figure 5.**
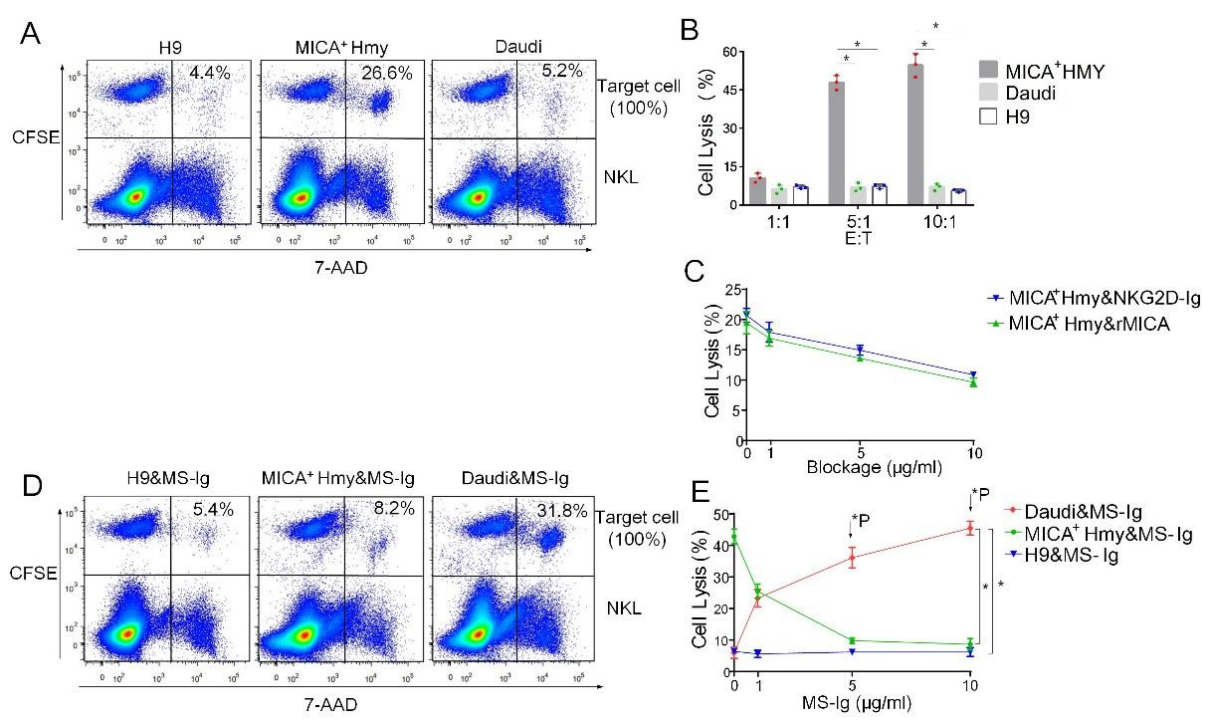
**MS-Ig mediates the cytotoxicity of NKL cells toward Daudi cells**. (**A**) Direct killing effect of NKL cells on target cells in the absence of MS-Ig. H9 cells, MICA^+^ HMy2.CIR cells, and Daudi cells were stained with CFSE, and NKL cells were added for 4 h of co-incubation. Apoptotic cells stained with 7-AAD. After flow cytometric analysis, the percentage of 7-AAD^+^ cells was calculated, with the CFSE^+^ target cells taken as 100%. (**B**) Percentage of target cells killed directly by NKL cells under different effector-to-target ratios. (**C**) Blocking effect of NKG2D-Ig and rMICA on the cytotoxicity of NKL cells toward MICA^+^ HMy2.CIR cells. (**D**) MS-Ig mediated the NKL cell killing of Daudi cells. MS-Ig was incubated with the target cells and NKL cells, and the percentage of apoptotic cells was evaluated using flow cytometry. (**E**) The MS-Ig-mediated killing effect of the NKL cells on the three types of target cells was verified using different concentrations of MS-Ig. *P < 0.05, based on the chi-squared test for panel B and the independent samples t-test for panels C and E.

**Figure 6.**
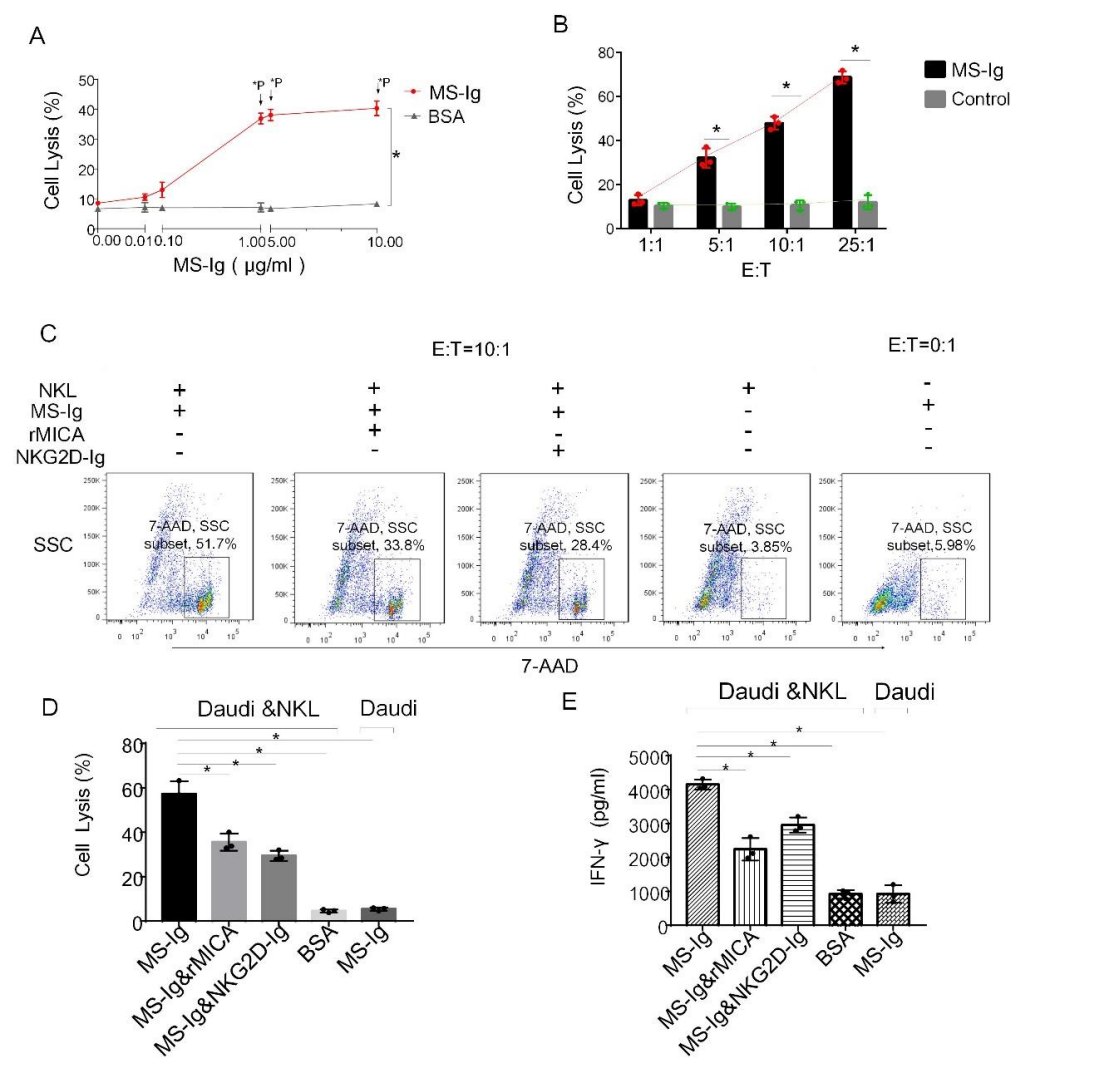
**MS-Ig-mediated killing is triggered by the binding between MICA and NKG2D to activate NKL cells**. (**A**) Killing curve of Daudi cells according to the MS-Ig concentration gradient tested. BSA protein was used as the control group. (**B**) Killing curve of Daudi cells at different effector-to-target ratios in the presence of MS-Ig. Daudi cells co-incubated with NKL cells in the absence of MS-Ig were used as the control group. (**C**) Flow cytometry results of the blocking effects of rMICA and NKG2D-Ig on the killing of target cells under fixed optimum killing conditions (MS-Ig concentration of 1 µg/mL and effector-to-target ratio of 10:1). (**D**) Column of the percentage of apoptotic target cells in panel C. (**E**) Column of the IFN-γ concentration in the cell culture supernatant under the same conditions as described for panels C and D. *P < 0.05, based on the chi-squared test for panels A, B, and D and the independent samples t-test for panel E.

### Soluble MS-Ig can mediate the cytotoxicity of NKL cells toward target CD20^+^MICA^-^ cells

MS-Ig binds specifically to CD20^+^ Daudi cells, as if installing a target of NK cells on the cancerous cells. To determine whether soluble MS-Ig acts as a bridge to mediate the specific killing of target cells by NKL cells, the H9, MICA^+^ HMY2.CIR, and CD20^+^ Daudi cell lines were used as target cells, respectively. The live cells were first stained with CFSE before being co-incubated with NKL cells at an E:T ratio of 10:1. Then, the percentage of apoptotic target cells (CFSE^+^7-AAD^+^) was determined using flow cytometry to evaluate the killing effect. In the absence of MS-Ig and at the E:T ratio of 5:1, the NKL cells had a greater direct killing effect on MICA^+^ HMy2.CIR cells (26.6%) compared with that on H9 (4.4%) and Daudi cells (5.2%) (Fig. 5A). At the E:T ratio of 10:1, the efficiency of NKL cells in killing MICA^+^ HMy2.CIR cells was 55%, whereas that for the killing of CD20^-^MICA^-^ H9 cells and CD20^+^MICA^-^ Daudi cells was only approximately 8%, respectively. The percentages of MICA^+^ HMy2.CIR, Daudi, and H9 cells killed were 54%, 7%, and 6%, respectively, at the E:T ratio of 10:1, with the NKL cells displaying significant direct cytotoxicity toward the MICA^+^ HMy2.CIR cells (χ^2^ = 42.1, P < 0.01) (Fig. 5B). When soluble NKG2D-Ig or rMICA was added to the MICA^+^ HMy2.CIR and NKL cell reaction system (E:T = 3:1), the percentage of target cells killed decreased from 20% to 10%, suggesting that the direct cytotoxic effect of the NKL cells activated by the interaction between NKG2D and MICA had been blocked (Fig. 5C). When soluble MS-Ig (5 μg/mL) was added to the cell reaction systems, there was no difference in the killing rate of H9 cells (4.4% vs. 5.4%) compared with the H9 system without MS-Ig added (Fig. 5A), but the killing rate of MICA^+^ HMy2.CIR cells had decreased from 26.6% (system without MS-Ig) to 8.2%. Notably, the killing rate of Daudi cells had increased from 5.2% (system without MS-Ig) to 31.8%, with the proportion of cells killed increased to 46%, whereas the killing rate of H9 cells was only 9% at the MS-Ig concentration of 10 µg/mL, which showed the significant killing effect of NKL cells on Daudi cells (χ^2^ = 36.6, P < 0.01) (Fig. 5D, Fig. 5E). The dose-effect relationship between the MS-Ig concentration and target cell death rate was further investigated. At the MS-Ig concentration range of 0-10 μg/mL, the efficiency of NKL cells in killing CD20^+^ Daudi cells increased gradually with increasing MS-Ig concentration (Fig. 5E). By contrast, the MICA^+^ HMy2.CIR cell death rate decreased and there was no effect on the CD20^-^MICA^-^ H9 cells (Fig. 5E).

### MS-Ig specifically mediates the targeted cell killing effect of NKL cells through the NKG2D-NKG2DL axis

To confirm that the cytotoxic specificity of the NKL cells toward CD20^+^ Daudi cells is indeed mediated by MS-Ig, the killing effect over an MS-Ig concentration range of 0-10 μg/mL was tested. The maximum killing effect occurred at the MS-Ig concentration of 1 µg/mL (Fig. 6A) and this was therefore selected as the optimal concentration to use for killing target cells. Next, different E:T ratios (1:1-25:1) were tested to determine which effector cell amount would result in the optimum killing of CD20^+^ Daudi cells. The killing efficiency increased (15% to 68%) with increasing E:T ratio. At the E:T ratio of 10:1, the killing rate of Daudi cells had increased to 48%, whereas that of the control was only 10%, indicating the significant cytotoxicity of the NKL cells toward these CD20^+^ target cells (χ^2^ = 33.6, P < 0.01) (Fig. 6B). Next, the percentage of killed Daudi cells was analyzed in both the presence and absence of NKL cells, MS-Ig, rMICA, and NKG2D-Ig at the E:T ratio of 10:1. In the presence of NKL cells, 1 µg/mL MS-Ig, and 1 µg/mL of either rMICA or NKG2D-Ig, the killing rate decreased from 51.7% to approximately 30%. However, when BSA was added to the Daudi and NKL cell reaction system, or when Daudi cells were co-incubated with MS-Ig only, there was almost no killing effect observed (Fig. 6C, Fig. 6D). The concentration of IFN-γ in all reaction systems was detected simultaneously and was observed to have decreased from 4500 pg/mL to 2000-3000 pg/mL in the presence of rMICA or NKG2D-Ig. The concentration of IFN-γ in the system without any killing effect was less than 1000 pg/mL. In other words, the concentration of IFN-γ was the lowest in the non-killing group and was greater in the killing group than in the group with the competitive binding-blocking proteins (Fig. 6E). These results show that the NKL cell killing mechanism mediated by MS-Ig is based on its role as a bridge connecting the interactions between the NKG2D receptors on NKL cells and the CD20 molecules on the Daudi cell surface to trigger effector cell activation and achieve the specific killing of the target cells.

## DISCUSSION

For their use in the treatment of hematological cancers, CAR T cells and CAR NK cells need to be amplified in vitro [32-35] and genetically engineered to express the appropriate CARs [36-40], both processes of which are time-consuming and costly, not to mention that immune cell infusion can be toxic to normal cells in the body as well [41-44]. By contrast, a soluble NK-CAR has the following advantages: its single-chain antibody can be partially replaced by any tumor surface antigen antibody, which is flexible in application; it can be used as a drug without the need for personalized treatment; and it can reach all parts of the body via the blood circulatory system to recruit the body’s own NK cells for killing target cells, thereby reducing the toxicity caused by immune cell infusion. Moreover, the process for producing the soluble MS-Ig fusion protein is simple and less time consuming, saving on production costs to make the product conducive to clinical implementation [45].

Our novel soluble NK-CAR is tailor-made for NK cells, as the NKG2D receptor is unique to these cells and CD8^+^ T cells [46]. Once bound with its ligand, the NKG2D receptor can directly activate the NK cell. Using this theory, we designed a single-chain CAR carrying a specific antibody against a tumor cell antigen and labeled the NKG2DL molecule on the surface of CD20^+^ target cells to trigger the killing activity of surrounding NK cells. In a previous study, we had designed several different MS-Ig structures to optimize the VH and VL sequences of the single-chain antibody and Fc components, respectively. The single-chain antibody has the best effect when the VH and VL sequences are connected. The currently selected Fc sequence does not affect the functions of all parts of the CAR and allows the protein to be formed as a homodimer.

In this study, NKG2D-2B4 reporter cells were used, which brought the advantage of allowing the activation of the NKG2D receptor signaling pathway to be visualized in this study. The immobilized MS-Ig could activate NKG2D-2B4 cells, indicating that it had the ability to activate the NKG2D receptor signaling pathway. This study involved immobilized MS-Ig, because our previous study had indicated that soluble rMICA does not activate the NKG2D signaling pathway [31, 47], whereas target cells expressing membrane MICA molecules can activate NK cells and be killed. This was determined to be attributed to the binding of the membrane-immobilized MICA on the target cells with the NKG2D receptor on NK cells, producing an NKG2D receptor aggregate that could effectively activate the cytotoxic activity of the effector cells. Therefore, based on the findings of the previous studies, immobilized MS-Ig was used to activate the NKG2D reporter cells and NKL cells in this study.

MICA^+^ Hmy2.CIR cells were used as important control cells because they express membranous MICA molecules and can directly activate and be killed by NK cells. Therefore, their use allows a clearer comparison of whether the MICA attached to the membrane of target cells after the binding of soluble MS-Ig has the same ability to activate and mediate the cytotoxicity of NK cells toward target cells.

We have found that in the absence of MS-Ig, NKL cells had no killing effect on CD20^+^MICA^-^ Daudi cells, and in the absence of NKL cells, MS-Ig had no toxic effect on Daudi cells. It was only when MS-Ig bonded specifically to both the CD20 molecule on CD20^+^ Daudi cells and the NKG2D receptor on NKL cells—thereby acting as a bridge to connect these effector cells to the target cells—that the NKL cells were activated to kill the Daudi cells. The formation of the homodimeric form of MS-Ig, which is more stable than the monomeric form, is facilitated by its Fc component. Activated NK cells secrete a variety of cytokines, with IFN-γ being a major type [48, 49]. Because IFN-γ plays an important role in immune regulation in the killing process of NK cells, it can serve as a key indicator for evaluating the activation of these effector cells.

NK cells express both activating and inhibitory receptors [50-52] and are activated only when the signal for their activation is stronger than that for their inhibition. Many tumor cells express high levels of HLAs, which inhibit the killing effect of NK cells [53-55]. Therefore, although soluble NK-CARs have many advantages and good clinical application prospects, they will only be better used if these challenges presented by tumor cells are overcome.
